# The Book World of Medicine and Science

**Published:** 1897-09-25

**Authors:** 


### The Book World of Medicine and Science.

A System of Medicine. By Many Writers. Edited by Thomas Clifford Allbutt, M.A., M.D, LL.D., F.R.C.P., F.R.S., Regius Professor of Physic in the University of Cambridge. Vol. II. (London: Macmillan and Co.) 1897.

This volume admirably fulfils the expectations raised by the one which preceded it, and there can be no doubt that for some years to come "Allbutt's Medicine" will be the standard work to which students and practitioners will refer in regard to the wide range of subjects which are dealt with in its pages. The volume before us is mostly occupied in the consideration of infections and intoxications. The subject of tuberculosis is treated by Dr. Sidney Martin, who gives a full and careful description of the present state of our knowledge of the disease, its etiology, pathology, and symptomatology. It is a little disappointing to find treatment dismissed in seven lines, but, of course, the treatment of the special developments of tuberculosis in the various parts of the body is dealt with in other portions of the book.

The article by Dr. Phineas Abraham on leprosy is very good and enlightening in every way. It again is largely etiological and pathological, but both treatment and prophylaxis are fairly dealt with. The question of the compulsory segregation of lepers is discussed, and although it is admitted that, in Norway for example, leprosy has only diminished since measures with this object have been adopted, still he attributes much of the decline in the disease to improved sanitation, to the better food, and increased prosperity of the people. Segregation does not seem to have been carried out completely anywhere, and Dr. Abraham says that in most countries it is impracticable. It must, moreover, be remembered that "the contagiousness of leprosy, if it exist, is very slight, that its inoculation is extremely difficult to bring about, and that it is a somewhat rare event for persons in association with lepers to contract the disease."

The articles on the eruptive fevers are of great excellence, and will be found most useful by young practitioners, who are too often launched upon the world with but little practical experience in the management of these diseases. Dr. Dawson Williams, in treating of measles, lays especial stress on the importance of the secondary affections, many of which seem to originate from infective material grown or accumulated in the mouth or fauces. He advises the regular use of antiseptic mouth washes, and suggests a solution of boric acid (1 to 2 per cent.) for the purpose.

Dr. Caiger, in treating of scarlet fever, writes still more emphatically as to the urgent necessity of keeping the mouth and fauces clean. In mild cases he uses boracic acid, bicarbonate of soda, sulphurous acid, or Condy's fluid, but in septic cases with ulceration or commencing exudation more vigorous methods are called for. In such cases he uses an acid solution of chlorate of potash, containing a large amount of free chlorine. This he applies with four-ounce syringe, by means of which the fauces and nares are thoroughly syringed out every two or three hours. By this means the entire naso-faucial tract is cleared of its offensive secretions. "No amount of gargling, spraying, or swabbing can compare with it in point of efficacy, even if the points be reached. The patient's head should be held over a basin and the mouth kept open. The solution may be injected with some force, as no harm can be done by a stream of fluid; but sufficient time must be allowed for the patient to get a breath between each squeeze of the syringe. The relief obtained is very great." A very interesting article is contributed by Sir Joseph Fayrer on "The Climate and Some of the Fevers of India," a perusal of which leaves us very strongly impressed with the large field which this subject offers for research and differentiation. There can, we think, be very little doubt that from among the mass of pyrexial diseases met with in that and other tropical areas many separate specific fevers will by degrees be sorted out. The subject of vaccinia is very completely dealt with. Dr. Theodore Dyke Acland treats of "Vaccinia in Man: A Clinical Study," giving, in a most impartially-written article, a complete account of the disease and the various complications which sometimes occur in its course. He also deals with the questions which have arisen as to the possibility of other diseases being transmitted along with it, and with the means to be adopted to prevent the occurrence of such accidents. The article is illustrated with several very admirable woodcuts. Dr. S. Monckton Copeman then takes up the story, dealing with the pathology of vaccinia; after which there comes an article by Mr. Ernest Hart on "Vaccination as a Branch of Preventive Medicine," an article which is a fairly complete review of the statistical side of the question.

The subject of the intoxications is a large and interesting one, the study of which is important not only for its own sake but as illustrating many pathological processes in maladies of a very diverse nature. In the absence or, at any rate, the rarity of examples of grain poisoning in England we are apt to forget how important and serious a matter it has again and again proved itself to be. Professor Allbutt, who deals with this subject (as also with Mushroom Poisoning, Opium Poisoning, and Other Intoxications), shows how instructive are the parallels between the effects of grain poisonings and certain forms of spinal disease common among ourselves; and indeed, in view of much that we now seem to know about the toxic origin of apparently organic nervous disease, as after diphtheria, the history given of the very widespread effects of the bacterial poisoning of ergotism in former times and of pellagra at the present day are of great interest and importance. The list of diseases which we look upon as "trophic" is continually increasing, but at the same time it is being shown how largely the nervous degeneration on which these trophic changes depend is due to the selective action of certain intoxications on certain parts of the nervous system—a reversion to a modified humoral pathology which is very manifest at the present day. The article on "Metallic and Some Other Forms of Poisoning, Including Poisonous Trades," by Dr. Thomas Oliver, contains a good deal that is practically new to the text books. Various forms of trade poisoning by zinc, antimony, bisulphide of carbon, nitro- and dinitro-benzole, and aniline, by carbonic oxide, and by various explosives such as roburite, nitro-glycerine, gun-cotton, dynamite, blasting gelatine, and picric acid, are dealt with, besides poisoning by carbolic acid and by ordinary metals such as lead, copper, and arsenic. The last hundred pages are occupied by a series of well-illustrated articles on "Internal Parasites." Altogether, a most admirable volume, well written, well illustrated, well printed—and practical withal.

